# “None of Them Could Say They Ever Had Seen Them, but Only Had It from Others”: Encounters with Animals in Eighteenth-Century Natural Histories of Greenland

**DOI:** 10.3390/ani10112024

**Published:** 2020-11-03

**Authors:** Helen Parish

**Affiliations:** Department of History, University of Reading, Whiteknights, Reading RG6 6AH, UK; h.l.parish@reading.ac.uk

**Keywords:** animals, natural history, missionaries, observation, encounter, ethnography, authorial voice, social networks

## Abstract

**Simple Summary:**

This article uses eighteenth century natural histories of Greenland to engage with recent debates over the role of personal observation and encounter with animals in the history of natural knowledge. In particular, it draws upon the works of Hans Egede, David Cranz, and Otto Fabricius to explore the connections between missionary work, natural history, ethnography and cultural exchange in this period. It asserts the importance of a more nuanced understanding of histories of human encounters with animals and nature in the early modern period, and human encounters with that history.

**Abstract:**

The pages of early modern natural histories expose the plasticity of the natural world, and the variegated nature of the encounter between human and animal in this period. Descriptions of the flora and fauna reflect this kind of negotiated encounter between the world that is seen, that which is heard about, and that which is constructed from the language of the sacred text of scripture. The natural histories of Greenland that form the basis of this analysis exemplify the complexity of human–animal encounters in this period, and the intersections that existed between natural and unnatural, written authority and personal testimony, and culture, belief, and ethnography in natural histories. They invite a more nuanced understanding of the ways in which animals and people interact in the making of culture, and demonstrate the contribution made by such texts to the study of animal encounters, cultures, and concepts. This article explores the intersection between natural history and the work of Christian mission in the eighteenth century, and the connections between personal encounter, ethnography, history, and oral and written tradition. The analysis demonstrates that European natural histories continued to be anthropocentric in content and tone, the product of what was believed, as much as what was seen.

## 1. Introduction

The fifth chapter of Hans Egede’s natural history of Greenland (1741) includes a description *Of the Land Animals, and Land Fowls or Birds of Greenland, and how they hunt and kill them*. Amid the account of bears, reindeer, hares, birds, seals and insects, Egede inserted a reference to “another kind of ravenous Beast, which eagerly pursue other beasts as well as men.” Significantly, “none of them could say they ever had seen them, but only had it from others” [1,2]. The amarok, then, is a creature that everyone knows about, but which has never been seen. Egede was not convinced that the amarok existed. On the basis that “none of our own people, who have travelled up and down the country, ever met with any such beast”, he judged, “I take it to be a mere fable.” 

Egede’s account of the language, culture, and natural environment of Greenland was published in 1729 [3] as *The Old Greenland’s New Perlustration* (*Det gamle Grønlands nye Perlustration*) and greatly expanded in a 1741 edition as the *Natural History of Greenland*. [4] The pages of Egede’s text, and early modern natural histories, expose the plasticity of the natural world, and the variegated nature of the encounter between human and animal in this period. His account of the flora and fauna of Greenland reflects this kind of negotiated encounter between the world that he sees, that which he hears about, and that which is constructed from the language of the sacred text of scripture. The amarok is just one small part of this larger picture, but it provides us with a lens through which to view the multiple meanings of that encounter. Its appearance in Egede’s work exemplifies the complexity of human–animal encounters in this period, and the intersections that existed between natural and unnatural, written authority and personal testimony, and culture, belief, and ethnography in natural histories. Egede’s meeting with the amarok was neither physical nor ocular; it was a mental encounter with an invisible, even imagined, animal. The amarok was a locus of imagination and categorisation, a particular form of encounter that embodied the world-view that informed Egede’s work. As Susan Crane has observed, some such encounters in natural histories “attend to lived interactions and some are largely fantastic”, but a better understanding of the ways in which animals and people interact in the making of culture makes a vital contribution to the study of animal encounters, cultures, and concepts [5,6].

For that reason, animals have been described as a “necessary part of our reconceptualization of ourselves as human” with an ability to convey meaning, and reveal mindset, as well as furnish observational detail [7,8,9]. Early modern natural histories described what the authors saw and observed, but were also informed by what they had read, and what they heard. The history of nature, and the history of human encounter with it, was anchored in both the observation of flora and fauna and the human history that shaped these encounters. This was a polyvalent approach to the history of nature, one that exploited the permeable boundary that existed between history, nature, and context, and which invited an encounter with the natural world that was material, observational, and philological [10,11,12,13]. It was possible for physical encounter, mental encounter, and spiritual encounter to coinhere on the same page of natural histories as different strands within the same process of description. Any investigation of the ways in which humans have encountered and interpreted animals prioritises the human voice and text. 

The living animal that emerges from the pages of early modern natural histories can only impart meaning if that written narrative is situated within its mental, geographical, and conceptual context. Thus, Egede’s work, and other narratives of missionary encounters with nature, are mediated through the authorial voice, and the voices of those from whom knowledge was gleaned. The very process of selection and organisation of the flora and fauna described imposed upon the material a particular mindset and perspective, and the interpretative framework within which encounter was expressed. The process of description and interpretation was both individual and collective, conscious and unconscious. Such texts also illustrate the way that the voices of the past could be used to mediate in the encounter between human and animal in the present [14,15]. Egede’s natural history, and that of other missionaries to Greenland, provide an illustration of the connections between missionary impulse, ethnography, and the writing of natural history.

## 2. Materials and Methods

This article uses three natural histories of Greenland, each composed by European Christian missionaries, to explore the interactions of encounter, evangelisation, and ethnography, the authority of oral and written evidence, and the importance of network and community in shaping human-animal encounters. The three source texts lend themselves to a focused comparative analysis; their writing and publication is chronologically contiguous, their content similar enough to track subtle shifts, but sufficiently reflective of authorial priorities to enable us to observe the impact of personal interactions with the natural world in the pages of these encyclopaedic narratives. Via these case studies, this article will engage with ongoing debates over the interdependence of early modern natural histories, as a shared enterprise of knowledge production that was created not only by their writers, but by the interventions and expectations of their patrons. It argues for the complexity and variety of the encounters that inform the writing of natural histories, and the multifaceted nature of the narratives contained in the texts. Running through the analysis is a determined assertion of the value—even the necessity—of reading such texts in context, and appreciating their position at the intersection of religion, ethnography, natural history, antiquarianism and cryptozoology. To do so is to develop a better understanding of human perception of animals in this period, which should then encourage a similar recognition of the ways in which the science of nature and knowledge become “historical” in the borrowing of methods, ideas, and conceptual frameworks in narratives of human–animal encounters in the modern world [16].

As the anthropologist Claude Lévi-Strauss commented, animals are “good to think with”, providing human society with symbols that enable reflection of the natural of humanity in this world and beyond [17]. In that respect, an animal that was presented as a figment of the collective imagination may seem an unlikely starting point for such an analysis. However, there is good reason to accept that animals that occupied a space beyond the eye of human experience might also be good to think with. In this sense, the current study offers an invitation to engage in debate with not just histories of natural history, but also with the narrative frameworks of cryptozoology. Peter Dendle’s analysis of the significance of cryptids in the context of their historical analogues encourages an acceptance that “no age has been without its share of hidden creatures” but also an appreciation that the debates over the confirmed existence of such creatures have been part of the record of human knowledge for centuries [18,19]. Imagined or invisible nature could act as a locus for dialogue and interaction as much as conflict. Like the amarok, it can also become a lens through which we can better see the interleaving of natural history with other forms of knowledge creation. 

In some respects it has been ever thus. Pliny’s natural history was not simply a work of observation and description, but an encyclopedia of knowledge culled from the realms of personal experience, travel writings, ancient sources and myths [20]. In no sense did the personal experience trump the narratives of the ancients or the narratives of those who had travelled widely enough to return with stories of the unknown. The *Naturalis Historia* included as much as there was to know of the creatures that walked the earth and inhabited its oceans, whether their existence outside the text could be evidenced. But what form would such evidence take? Brian Regal’s presentation of cryptozoology prioritises the difference between creatures for which there is evidence, but which no one has ever seen, and creatures which are seen, but for which there is no evidence [21].The amarok is part of this longstanding tradition, but also an example of the fluidity of the intersection between these two categories, as a creature that has never been seen, and for which there seems to be no evidence, but which everyone seems to know, and whose existence resisted conventional proof. Rauch’s influential study of the sukotyro, the extinction of a creature that never existed, provides a useful framework within which we can explore the multifaceted nature of the unseen amarok. If, as Rauch argues, anecdotes do not make a science, they do at least construct the culture from which science is created. The imagined or the unseen creature enjoyed an enduring presence in the pages of natural histories, which in turn shaped the direction of human knowledge and understanding. [22]. The amarok, like the sukotyro, invites a more nuanced approach to the intersections of history, culture, and science in the pages of natural history and human encounter. The article asserts the vital need to recognise the reality of the imaginary animal in at least one plane; if not in biological form, such creatures were nonetheless real in social and cultural form, in the narratives, myths, and personal encounters with that culture. Knowledge of the natural world, this case study demonstrates, was not just experimental, but also historical and social [23,24]. The taxonomies of Gesner and (to some extent) Linnaeus enabled Egede and his successors to find a place for the amarok in their histories, in a form that was both recognisably informed by the oral cultures of Greenland, and recognisable within the languages of European natural histories and the nascent “science of describing” [15]. 

It is against this backdrop that this paper debates the representation of human–animal encounters in early modern natural histories. The terminology of encounter in such texts is imbued with linguistic, geographical, textual and historical influences. From the presence of the amarok in Egede’s *Perlustration* we can already glean insights into the co-constitution of human and nonhuman interactions, and the visibility of the nonhuman in both metaphorical and material form. The multiple forms of intersection and interaction that emerge from these histories of nature enables us to map the ongoing processes by which knowledge was described and produced. Politics, culture, economic ambition, religious belief and personal observation interacted in the compilation of these descriptions of Greenland in a way that allowed not only the texts, but the broader context in which they were written, to be infiltrated by human interactions with animals, commodities and the forces of nature. An analysis of these sources demonstrates the extent to which human knowledge was—and is—the product of contingent historical processes. To describe Egede’s work as a form of multispecies ethnography may be stretching the point, but it is still vital that we recognise that those textual, mental and physical spaces in which the boundary between nature and culture are blurred are an essential part of the processes by which human and nonhuman encounters emerge, and shape each other [25,26,27,28]. The natural histories of Greenland are not simply exercises in cataloguing, I would argue, but texts and dialogues that were informed by the remarkable depth of knowledge that their authors acquired about the cultures and communities in which they lived and worked. The missionaries who compiled them were a pivotal part of the (global) circulation of knowledge in this period, and their written works evince the breadth and depth of their insights into cultures, language, scientific observation and historical contextualisation. Their writings mediate an encounter between observed and reported knowledge, ideas of the past and interpretations of the present, and the oral cultures of native populations through whom such information was shared. Part history, part natural history, part evangelism, and part antiquarianism, such knowledge production was the outcome of a dialectical relationship between culture, observation and memory, and between the missionary authors and their indigenous guides. The connections and the tensions between antiquarianism and natural histories, between material objects and the myths that surround them and between the immersive nature of observational field work and synoptic writing, expose the process of imbrication by which such knowledge was created [16,29,30,31]. 

## 3. Discussion

### 3.1. Writing the Natural History of Greenland

As parson to the isolated archipelago community of Lofoten, and self-styled apostle of Greenland, the Lutheran missionary Hans Egede developed a consuming interest in old Norse settlements on Greenland. In 1711 Egede sought permission from Frederick IV of Denmark to establish a Christian mission and colony in the territory, on the assumption that its people had once espoused a pure and primitive Christian faith that could be rekindled. Egede’s primary concern was the fulfilment of his missionary ambitions, and the restoration of a Christian church that would arrest the decline of the population into superstition and savagery [32]. However, the possibility of improved trading connections, and the exploitation of the economic value of Greenland, were from the outset part of the rhetoric of restoration and recovery that permeated Egede’s narrative [2,33]. Frederik IVs commitment to the dissemination of the gospel in the colonies was evident in his establishment of a Mission College, but it was the foundation of the Bergen Company, with associated trading rights, that provided Egede’s enterprise with a solid financial footing [32]. Egede’s missionary endeavour received the support of a king who was not only committed to evangelisation, but also seeking a means to understand and exploit local conditions so as to undermine Dutch dominance of whaling and trading in the Davis Strait. Proof of the existence of a historical, cultural, and physical connection between Greenland and Denmark was key to the enterprise. Egede was insistent that the focus of his missionary activity was the (re)conversion of Greenlandic Norsemen who had lost contact with the Christian church and degenerated into savagery. The plausibility of that argument was questionable, presumably to Egede himself given his research into the languages of Greenland. Nevertheless, the continued presence of Norsemen in Greenland was necessary before it could be considered part of Dano–Norwegian territory, and a state-sponsored mission be justified [34].

The search for the lost settlement of Austerbygd, and the restoration of Christianity to the region, supported Danish claims to Greenland and the surrounding seas that were justified by the assertion of longstanding cultural and historical connections between Denmark and the northern Atlantic. Egede’s history was broad in scope and rich in detail, laden with references to Inuit customs, beliefs and behaviours, but his mapping of the territory, its landscape, and natural resources was also vital to the development of economic and political connections with Denmark. The foundation of settler colonies on the west coast, the support given to Egede’s efforts to spread the Gospel, and the economic value of his narrative of encounters and observations were all part of the same story [32,35]. Helen Curry and James Secord have observed “natural history [developed] as a way of cataloguing novelties, charting unfamiliar territories and inventorying potentially useful resources” [36].

The fragility of the evidence for the existence, or the location, of the original Norse settlement was no deterrent; in 1721 Egede, his family, and forty other colonists set sail from Bergen, arriving in Greenland on 3rd July. Egede stayed in Greenland for 15 years, learning the Inuit language, observing and describing the peoples and cultures of the island, and evangelising and catechising in less than hospitable surroundings [33]. European weapons and boats were a poor match for the environment, and Egede’s descriptions of Inuit hunting practices were informed by close observation of equipment and techniques, and the skills required in hunting and fishing for food. His voyages along the west coast were driven by his conviction that it was here that the older Norse settlements were to be found. However, these journeys were also a process of exploration, which combined Christian mission with the collection of information about the flora and fauna of the territory [37]. The encounter with the natural world took place alongside the work of Christian mission; nature was a subject worthy of study in its own right, but it was also an implicit and an explicit channel for the communication of the divine will. Flora, fauna, and the very geology and topography of the earth imparted a message and knowledge that was simultaneously practical, pastoral, and providential. In this respect, the amarok reflects an ongoing process of negotiation between the world that Egede sees, that which he hears about, and that which is constructed from the language of authoritative texts. In the *Natural History of Greenland* [1], the visible and the invisible sit side by side, and the natural and unnatural, the material and the mythological, are encountered on the same page. Egede’s work embodied the encounter between his observation of land and ocean, and the challenge presented by the integration of the animals and sea creatures that inhabited the northern lands and waters into the authoritative taxonomies provided by Pliny’s Mediterranean world view. The *Natural History of Greenland* provided a detailed descriptions of flora and fauna, a juxtaposition of materials old and new into an encylopaedic account of the world around him, and a presentation of personal testimony and narrative, that anchored Egede in a different social and cultural network.

After Egede’s mission, the Danish Lutheran Church and German Moravian missionaries both sought to extend their influence over local Inuit populations, leading to a series of disputes between Hans Egede and Christian David, the driving force behind the Moravian missions. Forty years after Egede’s mission to Greenland, the Moravian Brethren missionary David Cranz arrived in the territory. Cranz had studied theology in Halle, before joining the Moravian Church in the early 1740s. On the instruction of Nikolaus von Zinzendorf, Cranz travelled to Greenland in 1761, with the intention of observing and gathering information about the peoples, languages, and beliefs of the indigenous populations, and the geography, geology, zoology and marine biology of Greenland. In what was essentially a hagiographic narrative for the Moravian church, Cranz was keen to assert the effectiveness of its activity, while at the same time avoiding any engagement with the rivalry between the two churches, securing patronage, and contributing to the exchange of knowledge of the natural world [34,38,39,40,41].

The *History of Greenland* that followed was a history of the Moravian mission, set within the context of a detailed account of the natural history of Greenland. In Cranz’s *History* [39], as Felicity Jensz has observed, the natural history preceded the history of the indigenous peoples; the “Genesis narrative created a continuity between nature and its ‘heathen’ peoples, whose ‘original’ state before their interaction with Europeans was to become debased through modern vices” [41]. There are echoes here of Egede, whose own history of Greenland was printed in German in 1763, as Cranz was preparing to publish his own work. The amarok described by Egede also featured in Cranz’s narrative, although he was no more convinced than Egede that the creature was real. “Some Greenlanders pretend to have seen black bears” he wrote “and their imagination aided by fear, have exaggerated them into monsters… but it is more usual among the natives to talk of a certain species of tiger, which they call Amarok.” These animals, as they were described, were the size of a calf, and covered in black and white spots, but “have never been seen by any European.” In Cranz’s view, the amarok was most likely a species of spotted bear, which “have been known” to cross the ice between Greenland and Iceland [39]. The *Narrative of the First Settlement made by the United Brethren on the Coast of Labrador* [39] asserted that the quadrupeds of Greenland, including black bears and wolves existed in greater numbers in Labrador. The skins of these animals were on show, “but the greatest curiosity was the hide of an animal which haunts the Greenlanders in their dreams. They have a name for it, the Amarok, and they tremble while they describe it. It is of a dark grey colour, about the size of a large dog” [39].

Shortly after Cranz’s mission, Otto Fabricius arrived on the southwestern coast of Greenland. Over the next five years, Fabricius—like Egede and Cranz—engaged not only in evangelisation, but the observations and collection of zoological information. After his return to Denmark, Fabricius published his Latin *Fauna Groenlandica* (1780) [42] in which he described in detail some 473 animal species, including 132 vertebrates and 341 invertebrates, providing information about habitat, as well as local names, hunting practices, and the use to which each species was put. Fabricius’ descriptions were painstaking and based upon his close observation of human custom and animal behaviour. But this prioritisation of personal encounter was not exclusive; like Egede and Cranz, Fabricius immersed himself in local custom and culture, and drew upon its lore and lexicon to inform and shape his own categorisations. Thus, under the heading “ursus luscus”, he added the amarok, which he described as a fierce wolverine, echoing the words of Egede that although everyone he met know of the amarok, and feared the creature, it had never been seen. The amarok was the “terror” of Greenland, “cuius simili tantum viso fugiunt, nec cito in locum talem veniunt. Indiuidua tamen paucissima dari verosimile est” [2,42]. It is worth noting that Hans Egede had been a family friend to Fabricius, making it likely that it was through Egede that Fabricius first encountered the combined fruits of natural history, missionary endeavour, and ethnographic study. In this respect, the intersection between these three histories of Greenland printed in the mid-eighteenth century provides insights into the way in which missionary encounters with the territory were informed by the social and cultural networks and exchanges that shaped the relationship between natural history and human history in this period [12,36,43,44]. Even those who sought to encounter, observe, and gather together the natural world while experiencing isolation and distance were engaged in a process of writing and communication that was moulded by networks of transmission [14,15].

### 3.2. Apprehending the Unknown

Robert Paine, in a contribution to the quincentenary of Columbus’ voyages, suggested that “the most intriguing question for anthropology coming out of the scholarly literature of that occasion, namely how, in the West, the *unknown is apprehended, then and now”* [35,45]. The explanation and classification of the unfamiliar in nature was often determined by an ability to establish connections with something that was known and understood. Fabricius’ study of the fauna of Greenland explicitly included animals that he had not seen, but for which he saw no good reason to assume that they could not have existed in Greenland. Oral testimony and lived experience entered the lexicon of description, but accounts of the creatures that inhabited the land and the sea still blended examples and knowledge culled from Pliny, Heliodorus, and biblical texts into this catalogue of personal observations [46]. Examining and touching the food consumed by whales in the waters around Greenland, Egede observed that “here we ought to praise the wise and kind providence of an Almighty Creator, who has made such mean things suffice for the maintenance of so vast an animal” [1]. Detailed descriptions of flora and fauna blended the old and new, expanded to include personal encounter. The communication of knowledge occurred in the personal presence of the author in the encounter and in the text. 

Animals have long been the “ubiquitous other” of human history. The observation and interpretation of nature, and the ordering of the natural world has been human preoccupation that has defined who we are for centuries [5,47,48]. Human–animals interactions possess a cultural, social and economic purpose that links past and present. The reading of nature, and human encounters with it, is both socially contingent and corrosive of the boundaries between natural history, religion, economic ambition, identity and ethnography. For that reason, natural history is rarely the history of nature alone. Natural knowledge was refracted through the lenses of theology, medicine, botany, philosophy, astronomy and geology, creating a narrative of encounter with the natural world that extended well beyond the description of flora and fauna. Natural history was a form of inquiry in which human interactions continued to loom large. 

In many cases—and the writings of missionaries who travelled to Greenland are a case in point—the process of collecting and cataloguing information was anchored in individual activity and experience, and which relied upon personal encounters with local populations, farmers, hunters and sailors. The study of nature might have been an international enterprise, channeled through networks created by shared interests, oral and written communication, and institutions of church and state, but it could never be distanced from the personal and regional context and cultures. Such influences permeate its pages, and the readers’ responses. In Egede’s *Perlustration* [4], the detailed observational accounts of local flora, fauna, and human encounter and exploitation were accompanied by an equally detailed narrative of his own alchemical activity. Egede’s acquisition of information about the natural resources of Greenland infused his accounts of experiments with alchemy, but that same process by which personal knowledge was obtained and exploited also turned the *Perlustration* into a visual and textual map of the materials that could be exploited by colonists, traders, and the Danish crown [49,50]. The third chapter of the book [4] *The nature of the soil, plants, and minerals of Greenland* presented historical and literary evidence for the fertility of the land, painting a picture that was then qualified by the process of personal observation. “We are informed by ancient histories” Egede wrote, “that the Greenland colonies bred a number of cattle, which afforded them milk, butter, and cheese in such abundance, that a great quantity thereof was brought over to Norway, and for its prime and particular goodness was set apart for the King’s kitchen…” Such histories also implied an abundance of wheat, corn, and fruitful trees. However Egede qualified this assertion based on personal experience: “all that has been said of the fruitfulness of the Greenland soil is to be understood of the latitude of 60° to 65°, and differs according to the different degrees of latitude. For in the most Northern parts you find neither herbs nor plants; so that the inhabitants cannot gather grass enough to put in their shoes to keep their feet warm, but are obliged to buy it from the Southern parts” [51]. By comparison, Chapter 17 [4] *Of the Greenland Trade, and whether, in promoting it, there is any Advantage to be expected* was couched in a more optimistic tone; if the old lands, formerly inhabited and manured by the Norway colonies, repopulated with men and cattle, Egede claimed, “they would, without doubt, yield as much as either Iceland or Feroe.” As far as trade was concerned, “if we once became masters of this trade, as it in justice belongs to us” it would be as profitable as any [49,50]. Egede was prepared to use personal encounter to criticise traditional assumptions, and to make a direct appeal to the interests of his patrons.

From Egede’s more detailed account of a “most dreadful” sea monster in the *Description of Greenland* we can glean deeper insights into the process by which knowledge of such creatures was constructed, processed, and presented in his work [1]. Egede had observed, described, and in some cases sketched, large sea creatures in the same waters. He listed and detailed eight types of whale and was clear in his distinction between whales and fish, “properly so called.” Yet in this particular instance, Egede believed that he had encountered something different. Through the printed text, he was familiar with the sea-creatures and monsters that featured in Tormoder’s History of Greenland, but had yet to encounter any of them in the northern seas, until this “terrible sea creature which in 1734 was seen in the sea outside the colony at 64 degrees.” Egede took care to provide as much tangible detail as possible, from the location of the sighting, to a description of the size and shape of the creature. The language used to describe the “monster” is revealing, reflecting Egede’s concern to provide reference points that would be familiar to his reader. The creature was large enough that its head “reached the yard arm and the body was as thick as the ship and was 3 to 4 times as long.” Its skin was wrinkled and rough, and the nose “long and pointed” and “blew like a whale.” At the rear, it was formed “like a serpent” and when it raised its tail from beneath the water, the tip was “a ship’s length” away from the body. A recent analysis of the text concludes that the sighting was a cetacean, but for our purposes modern debates over taxonomy are perhaps less revealing than the manner in which Egede presented his narrative [52]. His language, for example, was laden with comparisons between the familiar and the unfamiliar in a way that would support the external reader in deciphering and imagining the scale of the creature (like a whale, like a serpent, reached the yard arm.) However, if the reader was encountering the creature through Egede’s visual and verbal lens, Egede himself was writing in the context of what he had read, as well as what he had seen. Sea serpents of the type that he described were not unique to Egede’s work. Olaus Magnus, in the *Historia de Gentibus Septentrionalibus* (1557) [53] provided a detailed description of a 200-foot long Orm, living off the coast of Bergen. Unlike Egede’s report, this was not an eye-witness account, but one that was formed from the consolidation of evidence received from the local population and from sailors. Egede would not have been unaware of such narratives, and the monster that he recorded in the seas around Greenland can be seen as part of an ongoing process of concatenation of information and evidence from oral and written sources. 

Cranz’s natural history of Greenland [39] was likewise both a description of human encounters with animals, and a mechanism for encouraging further support and patronage for the missionary endeavour. The *History of Greenland* [39] was anchored in the experience of Christian mission and evangelisation, but it was far from being a devotional or theological text. The natural, social, and religious environment in Greenland were interdependent in Cranz’s mind, and in the historical narrative that provided the structure for his work. For both Egede and Cranz, the story of primitive Christianity in Greenland, and the erosion of that faith over the centuries, fuelled the missionary endeavour but also justified the settlement and exploitation of Greenland’s natural resources [41,54]. Cranz opened his history with a wide-ranging description of the natural environment, creating a verbal landscape that was only later populated with the language and culture of the indigenous people. The necessity and vitality of the missionary effort was woven into that history, but Cranz’s natural history also carried a political and economic value as a record of natural resources, social structures, and cultural forms. Setting aside any concerns about the devotional content of the book, secular trading societies started to view the Moravians as potential collaborators in other geographical regions, including the Danish settlement on the coast of Guinea [41]. Natural knowledge, formed out of the personal encounter with flora and fauna, readily became commercially profitable and politically useful. Collected specimens, information about farming, hunting, or mining practices, indigenous mental frameworks were shared, recorded and catalogued as part of the writing of natural history and missionary narratives. However, conversation could readily become corruption, as travellers, missionaries, colonists and traders sought to understand and exploit traditional human/non-human interactions in the search for not just knowledge, but for profit. 

### 3.3. Voices of Encounter

Observation and description defined, and was defined by, human encounters with that same nature, and with each other. One of the striking features of these natural histories of Greenland is the audibility of the authorial voice, and the voices of indigenous populations (albeit mediated through the lens of the missionary) and the dialogue between past and present [55]. As Clifford Geertz recognized, culture is the outcome of a layering of intertwined symbols and signs. In a comparative reading of the natural histories of Greenland, we can start to peel back some of these layers, and use the detailed narrative of personal experience that they contain to expose the patterns of cultural and social relationships in the context that produced such histories [56,57,58]. Such an approach recognises the existence of what Dendle describes as an enduring need in human society for “folkloric monsters” into which fears are projected, and through which human qualities are articulated or repudiated. Such mental constructs run through myths, proverbs and legends, but are also present in ritual actions (both secular and religious) and in the construction of cultural, social, and imagined identities [18,59]. Within the context of the eighteenth-century natural histories of Greenland the voices of encounter in human memory are memorialised in the written record. The existence of the amarok in the culture of the Inuit is asserted—albeit with some skepticism—by Egede, Cranz, and Fabricius. Nevertheless, its telling is a reflection on ethnographic reality, rather than a reflection of it [17] The amarok is far from a major actor on the stage of natural history, and the rather cursory treatment that it receives by Egede and others conceals, or at least diminishes, the more complex presence of the creature in Greenlandic myths investigated by Hinrich Rink in the mid-nineteenth century [60]. In the detailed retelling of the story of the amarok, it is clear that those who pressed the creature into their narratives of natural history did so without encountering, or engaging with, the cultural meaning that gave life to the amarok. 

In Rink’s telling of the myth of the orphan boy Kagsagsuk, the child heads to the mountains in order to seek the strength to escape from his poverty, and the bullying of his peers. Standing between the mountains, Kagsagsuk called out to the “Lord of Strength”, who appeared in the form of a large animal, the amarok [60]. The beast threw the boy to the ground, causing small bones to fall from his body. Each day Kagsagsuk returned to the amarok, more bones fell from his body and he grew in strength. Multiple variants of the narrative describe much the same story, with the role of the mediator played by the amarok, a grey wolf [59]. However, in a different legend, again retold in Rink’s *Tales and Traditions of the Eskimo*, [60] the role of the amarok was more sinister. After reports that an amarok had been “heard roaring” in the Godthaab, a man set off “to encounter the beast” accompanied by a relative. Coming across the amarok’s young, he killed them. The amarok returned, clutching a reindeer between its jaws, and searched for its young. Unable to find them it rushed to the lake, and pulled out a human form, at which point the hunter fell to the ground, his soul removed from his body by the amarok [60]. Similarities between the stories, particularly in the description of the size and shape of the amarok cannot mask the very different purposes to which the amarok is put, as the means by which an imputed moral meaning is imparted to the audience. In much the same way, the amarok provides a useful illustration of the malleability of a constructed but unseen creature, embedded in a regional oral culture but woven into the fabric of natural history devoid of its meaning. As Boria Sax has suggested, the question of whether an animal is real or imaginary is at the most basic level a dimension of taxonomy [23]. Imaginary animals may be the construction of the human mind, but they are often imbued with the tangible properties of the real. As a result, the boundary between the real and the unreal is both mobile and permeable [23]. For that reason, the form and content of written natural histories cease to make sense if they are removed from the mental and oral space inhabited by the creatures that they describe. 

What we learn from these texts reaches far beyond the flora and fauna of Greenland, into the mentality and motivations of the author, the interaction between the oral and the written, and the role of social contact in shaping the way that missionaries studied and encountered nature. The central process of description was modelled by the authors’ interactions with their subject, and by the local context, people, language, legends, and networks of transmission. Observational encounter was punctuated by local language and culture, textual sources, and physical experience [14,15]. 

For the Christian missionaries, an understanding of the territory in which they sought to work was formed at the intersection of previously acquired information about conquest, conversion, and culture, and the knowledge that was shared by indigenous peoples. The models of human/non-human encounter, classification, and categorisation in European natural histories arrived in Greenland with the missionaries, who superimposed these world views on the culture and landscape that they found. Flora and fauna might be slotted into such schema easily enough, but a full understanding relied on personal encounter, alongside engagement with local language, life, and custom [32]. The history of nature was embedded in the nature of history and antiquarianism; the same intellectual tools, methodologies and practices were common to the early modern study of nature and of the past [13,61]. However, this created a narrative of the past that connected Greenland with Europe that came under pressure from the lessons of the present, not least the ability of indigenous populations to live, hunt, and exert some kind of control over the natural world. European encounters with nature were formed alongside encounters with people, with the result that the observation and description of the natural history of Greenland was not simply an exercise in recording and categorisation, but an exercise in invention, instruction, and interpretation. 

Christian missionaries acquired and imparted a deep knowledge of the communities and cultures in which they lived, and their histories of human encounters with animals made a significant contribution to ethnographic writing [30,31]. An understanding of other cultures was a necessary precondition for the success of any missionary activity. Linguistic study and historical research often provided the means by which the Christian message was communicated, and were vital to its impact. Such unrivalled access to oral and written cultures fuelled cultural exchange, and produced narratives and descriptions that were simultaneously historical, ethnographic, religious and zoological. By becoming intimately involved with the new cultures that they both observed and evangelised, missionaries were capable of absorbing but also appropriating natural knowledge [62]. Thus, Egede’s *Perlustration* [3] included descriptions and depictions of Inuit hunting practices and tools, informed by his observations, and by encounters with fishermen and hunters from which he gleaned practical information and insights into the customs and stories of the region. Reindeer, he explained, were hunted throughout the summer season, with men accompanied by women and children and who startled the deer by clap-hunting. Where the number of people was insufficient to surround the herd, “then they put up white poles (to make up the number that is wanted) with pieces of turf to head them, which frightens the deer” [3]. Inuit hunting methods were “peculiar to themselves and their country” but Egede was swift to point out that they served the needs of the community well. When hunting whales, for example, “they put on their best gear or apparel, as if they were going to a wedding feast, fancying that if they did not come cleanly and neatly dressed, the whale, who cannot bear slovenly and dirty habits, would shun them and fly from them” [3].

Cranz’s *History of Greenland* [39] also presented detailed accounts of Inuit hunting, including an eye-witness description of the multiple types of dart used in hunting, and a narrative account of the various methods use to hunt seals in water and under the ice. Seals were driven under water by hunters clapping, shouting, and throwing stones, and without respiration were driven to the surface where they lingered in plain sight to recover [39]. Such descriptions were anchored in Cranz’s encounters with animals during the hunt in the present, integrated into a reading of the past in which the history of Norse settlements in Greenland provided an ancestry for the eighteenth-century inhabitants. Cranz made use of oral testimony when framing his descriptions, but clearly did not take all such information at face value, and was prepared to reject information that he found implausible. In the general overview of the territory with which his book opened, for example, Cranz speculated whether Greenland might be an island, or somehow connected to another continent. Dutch and Russian explorations had revealed that this was not the case to the east, but Cranz believed there to be good reason why there might be a connection to North America. However it was Cranz’s own analysis of the available evidence that took primacy; “to these reasons we may add the testimony of the Greenlanders themselves” he wrote, “though not much to be relied upon” [39].

Otto Fabricius’ missionary narrative and zoological writing was likewise punctuated by ethnographic observation. Like Egede and Cranz, Fabricius lived among the indigenous population, participating in hunting expeditions which he then described in detail. Such encounters provided Fabricius with a well-developed understanding of Inuit hunting techniques, but also the uses to which animals (including the polar fox, sperm whale, and humpback whale) were put. Such ethnographic information contributed to future attempts at evangelisation, but the recording of knowledge also shaped the writing of natural histories, enabling the interpolation of personal experience and interactions into descriptions of human contact with the natural world. Fabricius had travelled to Greenland with a copy of Linnaeus’ *Systema Naturae* [63] but in the *Fauna Groenlandica* [42] fairly swiftly rejected Linnaeus’ taxonomy. Instead, Fabricius based his system of classification on his close acquaintance with the Greenlanders. The summary of the seals found in the waters around Greenland was dominated by hunting narratives, which Fabricius punctuated with detailed descriptions of his participation in the hunt which was so vital to the survival of the population. After learning how to manage a kayak, Fabricius was then able to “accompany them on hunts of all kinds and there see, indeed learn for myself.” As a foreigner who could demonstrate some proficiency in hunting, “I, who could accompany them on this hunt, am told, that, even after so long a time, my memory is held in esteem by them” [35,42]. 

The works of Egede, Cranz and Fabricius demonstrate the extent to which the writing of natural history was a collaborative and collective enterprise, and one which exploited indigenous knowledge and informants alongside European authoritative texts [64,65,66,67]. It was precisely this kind of physical and verbal encounter with animals that enabled the inclusion of evocative descriptions in these natural histories, which themselves invited a questioning of preconceived ideas. Hans Egede drew upon the “relation of the whale-catchers” in his description of the hunters’ encounter with a whale, but also used his own encounter to question whether “such a vast body should need many smaller fishes and sea animals to feed upon.” On the contrary, Egede argued the whale’s food (*pulmo marinus*) had the appearance of blubber, dark brown in colour, and was moved around by the whale so slowly that “one may easily lay hold of it, and get it out of the water. It is like a jelly, soft and slippery, so that if you crush it between your fingers you find it fat and greasy like train oil” [3]. The incorporation of such observations, and the information provided by indigenous peoples, had a transformative effect on the writing of natural history. As Iris Sobrevilla has argued, “not only is it a history of nature in narrative form, as in the Plinian tradition; it is a history where nature, in the form of an animal, plays the main role in the history of a people” [67]. 

## 4. Conclusions

Just as the very process of observation, and the experience of encounter, was coloured by the preconceptions of the author, so was the process of inventory and interpretation. As Keith Thomas reminds us, the practice of observation was anchored in the pre-existing mental categories through which the observer encounters, orders and classifies the familiar and the unfamiliar [68]. Once learned, such compartmentalisation colours the way in which the world is seen, and conditions our behaviour within it [15,68]. As a result, the values that underpin human society and activity are implicitly projected onto the natural world, shaping encounters with animals, and exploiting nature as a means by which human values can be embedded or eroded. Influenced by Aristotle, the classification of animals was based on anatomy, habitat, and reproduction, but also their utility to man, their importance as food, and their ability to impart moral meaning. It would be misleading to suggest that by the mid-eighteenth century, European natural histories had ceased to be anthropocentric. The surveys of Greenland that were written by Egede, Cranz and Fabricius [3,39,42] might have taken topography as their starting point, but in all three cases the collection, categorisation, and presentation of the information was profoundly human in content and tone. These were texts in which the voice of the author, and human interaction, loomed large. Encounters with animals were presented in human terms, using human examples. The political, economic and religious context in which missionary activity evolved was woven into the descriptions of the natural world, shaping both the narrative, and the encounters within it [15,69]. 

Throughout the descriptions of Greenland, it is possible to observe the complex relationship between mentality, encounter, and description. The encounter with the amarok, with which we started, was a mental rather than a physical process, grounded in local legend and oral culture, but recorded in the written descriptions of the missionaries. Its value is more than anecdotal; that same process by which memory and mentality intersected with the materiality of nature is evident throughout these three texts in the references to “ancient histories”, in the narrative of a longstanding political and cultural connection between Europe and Greenland, the assertion that primitive Christianity had once thrived in the territory. Imagining and re-imagining remains a pivot in the histories of human encounters with animals and nature in the early modern period, and human encounters with that history. Of the amarok, Egede wrote that “none of them could say they ever had seen them, but only had it from others.” Seeing may be believing, but how animals were seen, remembered, and recorded, is still profoundly affected by human belief.
